# Small vessel disease and intracoronary plaque composition: a single centre cross-sectional observational study

**DOI:** 10.1038/s41598-019-39989-3

**Published:** 2019-03-12

**Authors:** A. Wightman, P. Barlis, M. MacBain, L. Hodgson, L. Cheng, S. Gocuk, U. Hayat, D. Chow, M. Tacey, A. Hutchinson, D. Colville, E. Lamoureux, J. Savige

**Affiliations:** 1 The University of Melbourne, Melbourne Health, Parkville, VIC 3050 Australia; 2 The University of Melbourne, Northern Health, Epping, VIC 3076 Australia; 3 The University of Melbourne Department of Ophthalmology, Royal Victorian Eye and Ear Hospital, East Melbourne, VIC 3101 Australia; 4 Northern Health, Epping, VIC 3076 Australia; 5 The University of Melbourne, Parkville, VIC 3050 Australia

## Abstract

Cardiac events are commonly triggered by rupture of intracoronary plaque. Many studies have suggested that retinal small vessel abnormalities predict cardiac events. The present study examined retinal microvascular abnormalities associated with intracoronary plaque. This was a single centre cross-sectional observational study of consecutive subjects who underwent coronary angiography and intracoronary optical coherence tomography (OCT) of occlusive coronary artery disease. Subjects’ retinal images were deidentified and graded for microvascular retinopathy (Wong and Mitchell classification), and vessel calibre using a semiautomated method based on Knudtson’s modification of the Parr Hubbard formula. Control subjects had no significant plaque on angiography. Analysis used the Fisher’s exact test or student t-test. Thirty-two subjects with intracoronary plaque including 22 males (79%) had a mean age of 62.6 ± 9.4 years. Twenty-four (86%) had hypertension, 10 (36%) had diabetes, and 21 (75%) were current or former smokers. Their average mean arterial pressure was 90.5 ± 5.8 mm Hg, and mean eGFR was 74 ± 15/min/1.73 m^2^. On angiography, 23 (82%) had a left anterior descending artery (LAD) stenosis, their mean diseased vessel score was 1.86 ± 1.21, and mean total stent number was 1.04 ± 1.00. Plaque type was mainly (>50%) fibrous (n = 7), lipid (n = 7), calcific (n = 10), or mixed (n = 4). Control subjects had a lower mean diastolic BP (p = 0.01), were less likely to have an LAD stenosis (p < 0.001), a lower mean diseased vessel score (p < 0.001) and fewer stents (p = 0.02). Subjects with plaque were more likely to have a moderate microvascular retinopathy than those with none (p = 0.004). Moderate retinopathy was more common with lipid (p = 0.05) or calcific (p = 0.003) plaque. Individuals with calcific plaque had a larger arteriole calibre (158.4 ± 15.2 µm) than those with no plaque (143.8 ± 10.6 µm, p = 0.02), but calibre was not related to diabetes or smoking. Calibre did not correlate with plaque length, thickness or arc angle. Thus, subjects with intracoronary artery plaque are more likely to have a moderate microvascular retinopathy. Those with calcific plaque have larger retinal arterioles which is consistent with our previous finding of larger vessel calibre in triple coronary artery disease. Retinal microvascular imaging warrants further evaluation in identifying severe coronary artery disease.

## Introduction

Cardiovascular disease due to coronary artery atherosclerosis remains a leading cause of mortality and morbidity worldwide^1^. Ischemic events typically result from thrombus lodging on unstable intraluminal plaque and subsequent vessel occlusion.

Coronary artery plaque can be visualized directly at angiography using intraluminal optical coherence tomography (OCT)^2^. OCT provides a detailed assessment of coronary plaque, including type (fibrotic, cholesterol, or calcific), thickness, length and arc angle.

However there is also interest in non-invasively assessing the risk of coronary artery disease and cardiac events. Epidemiological studies suggest that microvascular retinopathy features and vessel calibre predict cardiac events. Moderate microvascular retinopathy changes are associated with an increased risk of cardiac events^3,4^. Retinal arteriolar narrowing strongly correlates with angiographically-demonstrated coronary artery disease^5,6^, and narrowed arterioles are associated with reduced myocardial perfusion^7^ and increased coronary calcification^8^ on cardiac MRI and CT scans. Conversely, larger retinal venular calibre predicts events in women in epidemiological studies^9^. Retinal venular dilatation also predicts stroke^10^, probably through cerebral hypoxia, endothelial dysfunction, hyperglycemia and inflammation^11–13^. In addition, functional changes in the microvasculature are also associated with increased cardiac events, probably through shared exposure to metabolic and systemic stressors, and endothelial dysfunction^14^.

Our recent study confirmed the association of retinopathy with coronary artery disease^15^. Retinal hemorrhage, moderate microvascular retinopathy and proliferative diabetic retinopathy were all associated with an increased number of diseased coronary arteries, and a higher Leaman score. Venular calibre was larger with triple vessel disease.

The association of retinopathy features and cardiac disease is not unexpected because vascular risk factors are shared by both the retinal microvasculature and the coronary arteries. Retinal vessels have the advantage of being visualized directly. Retinal small vessel changes include arteriovenous nicking, focal and generalized narrowing, haemorrhage and exudates^16^. Vessel calibre can be measured accurately from retinal images^17^. Smaller retinal arterioles are generally associated with increased age, male gender, hypertension, and renal impairment, and larger arterioles with diabetes, smoking, obesity and inflammation^12,13,18^. Calibre is not affected by medication^19,20^. However both arteriole and venular calibre are interdependent, and as one increases or decreases, the other changes too.

Our previous study demonstrated that moderate microvascular and diabetic retinopathy predict more severe coronary artery disease. However the likelihood of a cardiac event also depends on plaque type and this study examines whether retinal vascular features also predict intracoronary plaque type.

## Design, Setting, Participants and Methods

### Study design

This was a single centre, cross-sectional, observational study of all subjects who underwent coronary angiography and intracoronary plaque OCT for clinical indications over an 18 month period. Subjects were recruited regardless of whether they had stenting at the time of angiography. Controls with normal angiography were recruited over a one month period at the same time. Exclusion criteria were previous coronary artery stents, an incomplete procedure, or ungradable retinal images. Randomisation was not performed.

The primary outcome was to demonstrate a relationship between microvascular retinopathy including calibre, and the presence of plaque or plaque type. There were no changes to the study design after its commencement and no interim analyses.

This study was approved by the Northern Health Human Research Ethics Committee according of the Principles of Helsinki, all methods were carried out in accordance with the relevant guidelines and regulations, and all participants provided signed, informed consent.

### Study subjects

All subjects who underwent intracoronary OCT at a metropolitan teaching hospital (Northern Health) for clinical indications over an 18 month period were studied. In general, recruitment, data capture, and retinal imaging were performed at the admission for angiography, but five subjects were recruited at a separate clinic visit.

Subjects were assisted to complete a structured questionnaire that included demographic data (age, gender), and vascular risk factors (smoking, diabetes, hypertension). Hypertension and diabetes were based on self-reported physician-made diagnoses. A clinic BP was recorded using a Hg sphygmomanometer after 5 minutes’ rest.

Subjects underwent left heart catheterisation, coronary angiography and OCT by a cardiologist (PB) according to a standard protocol^2,21^. OCT pullback was performed at 20 mm/sec using a non-occlusive technique with concomitant injection of isoosmolar contrast (C7 OCT Console LightLab Imaging Inc, Westford, MA). The presence of current or previous significant left anterior descending artery stenosis (LAD, >70%), total stent number and diseased vessel score (>70% stenosis in any of the four major vessels) were noted.

Intracoronary plaque was then examined on an off-line review station by a trained reader, and classified as mainly (>50%) fibrous, lipid, calcific or mixed type based on its morphology^21^. Plaque was also assessed for: length (from the number of consecutive frames in which it was visible with each frame representing 0.2 mm); depth (the distance between the lumen and the leading edge of the plaque feature); cap thickness (the thickness of a cap over OCT-delineated calcium or necrotic core); thickness (the thickest distance between the inner and outer surfaces of the plaque component, valid only if the deeper margin is clearly visible); and arc angle (arc measured using the center of mass of the lumen as the angle point)^21^.

### Retinal imaging and grading

Subjects underwent retinal photography in a darkened room using a Canon CR5-45NM non-mydriatic 45° retinal camera (Canon, Japan) with a 10.1 megapixel EOS 40-D back or a Canon CR-DGI non-mydriatic retinal camera (Canon, Japan) with an 8.2 megapixel Canon EOS 30D back. At least two images were taken of each eye, with one focusing on the optic disc and the other on the macula.

Retinal images were used to assess microvascular damage by two trained graders using a standardised protocol for microvascular retinopathy^16^. Mild retinopathy was characterised by generalised arteriolar narrowing (arteriolar width <67% venular width), focal arteriolar narrowing, silver wiring, low arteriovenous ratio or any combination of these signs. Moderate retinopathy had the features of haemorrhage, cotton wool spots, hard exudates, or any combination of these.

Retinal vessel calibre was graded at the Centre for Eye Research Australia (CERA) using a standardized protocol^17,22^. Images were coded, and an expert grader measured the calibre of vessels (IVAN software program). The six largest arterioles and venules were selected, and the calibre measured using the revised formula of Knudtson based on the Parr-Hubbard formula^17,22^. Results were summarised as the central retinal artery equivalent (CRAE) and the central retinal venular equivalent (CRVE). This method was highly reproducible with high intra-class correlation coefficients^18^.

#### Statistical analysis

Features were compared using Fisher’s exact test or the student t test (STATA version 11.2 software, College Station, TX, USA). Statistical significance was set at p < 0.05 and trends at <0.10.

## Results

Thirty-two subjects had intracoronary OCT, but 4 were excluded because of ungradable retinal images. The remaining 28 subjects included 22 males (79%) and had a mean age of 62.6 ± 9.4 years (Table 1). Twenty-four (86%) had hypertension, 10 (36%) had diabetes, and 21 (75%) were current or former smokers. Their mean arterial pressure was 90.5 ± 5.8 mm Hg, and their mean eGFR was 74 ± 15 ml/min/1.73 m^2^.

**Table 1:** Clinical, angiographic and plaque characteristics of study subjects.

Clinical features	Subjects with plaque (n = 28)	Subjects without significant plaque (n = 10)	Mean difference (95% CI) p value
Age (mean ± SD, years)	62.6 ± 9.4	65.8 ± 9.5	−3.2 (−10.2 to 3.8) p = 0.36
Gender (male)	22 (79%)	10 (100%)	**p = 0**.**16**
Hypertension	24 (86%)	5 (50%)	**p = 0**.**04**
Diabetes	10 (36%)	0 (0%)	p = 0.04
Current/past smoker	21 (75%)	8 (80%)	p = 1.00
Mean arterial pressure (mmHg)	90.5 ± 5.8	86.1 ± 13.9	4.4 (−2.0 to 10.8) p = 0.17
Systolic BP (mmHg)	123.3 ± 11.2	124.0 ± 16.9	−0.7 (−10.3 to 8.9) p = 0.88
Diastolic BP (mmHg)	73.6 ± 6.6	67.0 ± 13.4	6.6 (1.6 to 11.6) p = 0.01
eGFR (mL/min/1.73 m^2^)	74 ± 15	73 ± 17	1.0 (−10.6 to 12.6) p = 0.86
**Angiographic features**			
LAD stenosis (>70% or stent)	23 (82%)	0	**P** < **0**.**001**
Mean diseased vessel score (4 vessels studied)	1.86 ± 1.21	0.1 ± 0	1.76 (0.98 to 2.54) **p** < **0**.**001**
Mean number of stents	1.04 ± 1.00	0 + 0	1.04 (0.4 to 1.69) **p** = **0**.**002**
**Predominant plaque type**			
Fibrous	7 (25%)	N/A	
Lipid	7 (25%)	N/A	
Calcific	10 (36%)	N/A	
Mixed	4 (14%)	N/A	
**Retinal features**			
Hypertensive retinopathy			**p = 0**.**04**
None	0	1 (10%)	
Mild	14 (50%)	9 (90%)	
Moderate	14 (50%)	0	
**Retinal caliber**			
CRAE (µm)	147.6 ± 17.9	143.8 ± 10.6	3.8 (−8.4 to 16.0) p = 0.53)
CRVE (µm)	213.0 ± 24.9	211.4 ± 10.1	1.6 (−14.9 to 18.1) p = 0.85

### Coronary angiography and plaque features

On angiography, 23 subjects (82%) had a current or previously-stented LAD stenosis, their mean total stent number was 1.04 ± 1.00 each, and mean diseased vessel score was 1.86 ± 1.21.

**Figure 1:**
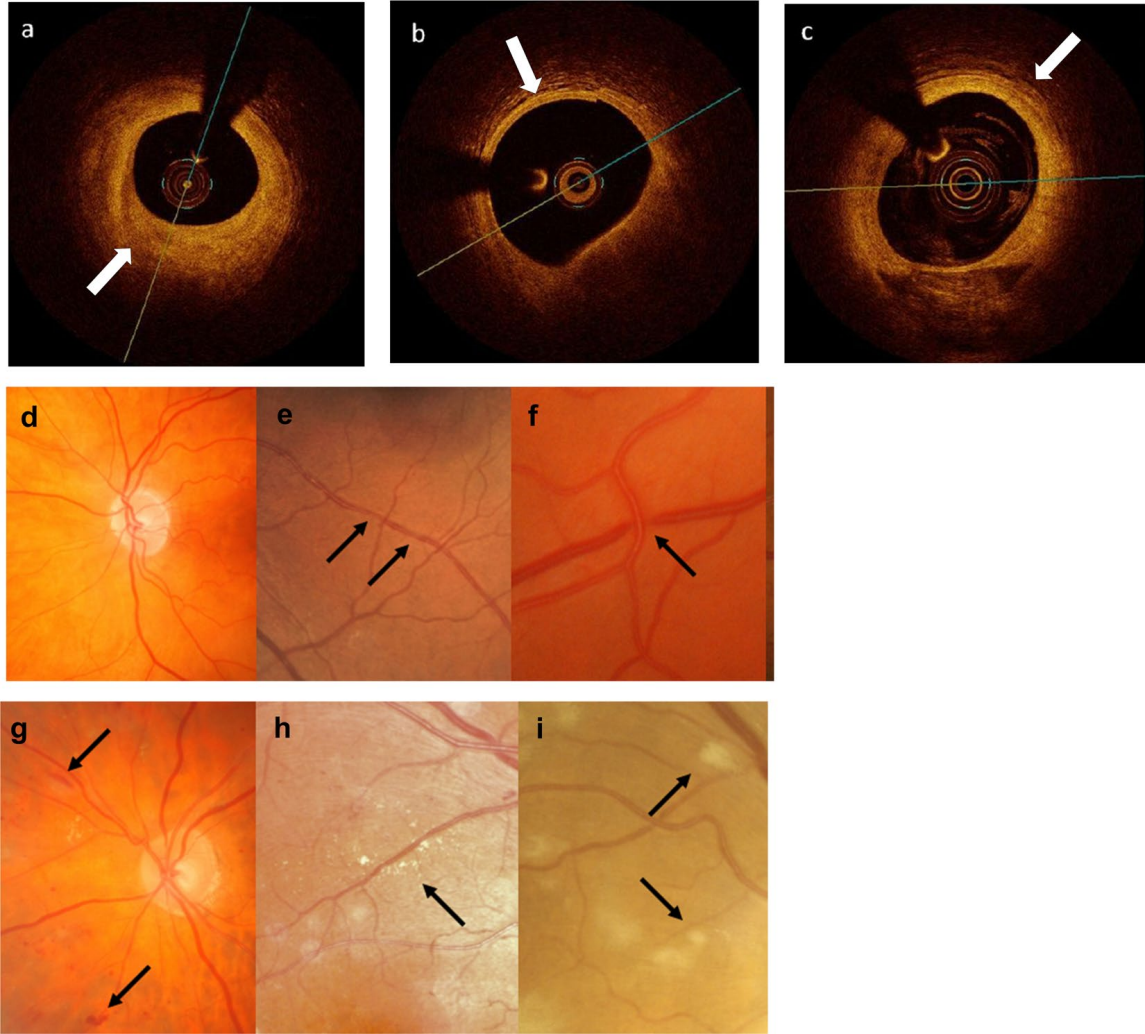
Plaque type (**a**–**c**) and retinopathy features (**d**–**i**). (**a**) Fibrous plaque; (**b**) lipid plaque; (**c**) calcific plaque; features of mild retinopathy: (**d**) generalized arteriole narrowing (arteriole <2/3 calibre of venule); (**e**) focal arteriole narrowing; (**f**) arteriovenous nicking (venule narrowed where crossed by arteriole); and features of moderate retinopathy. (**g**) Haemorrhages; (**h**) hard exudates; and (**i**) cotton wool spots soft exudates or infarcts.

Altogether the 28 subjects had 161 plaques, with a median of 4 (range 1–29). Seven subjects (25%) had mainly (>50%) fibrous plaque, 7 had mainly lipid (25%), and 10 (36%) had mainly calcific plaque (Fig. 1a–c). Four (14%) had mixed type plaque.

Control subjects with no significant stenotic plaque had fewer diagnoses of hypertension (p = 0.04) or diabetes (p = 0.04), a lower mean diastolic BP (p = 0.01), a lower likelihood of an LAD stenosis (p < 0.001), a lower mean diseased vessel score (p < 0.001), and fewer stents (p = 0.002).

### Comparison of clinical features in subjects with intracoronary plaque and none

When the features of subjects with fibrous, lipid or calcific plaque were compared, their mean age increased from fibrous plaque (56.3 ± 9.6 years) to lipid (63.1 ± 7.4 years) to calcific plaque (68.4 ± 9.4 years) (mean difference between calcific and fibrous, 12.10 (I 2.14 to 22.06) p = 0.02) (Table 2). Each study group comprised mainly men, with at least 80% with hypertension, and 71% who were current or former smokers. Individuals with calcific plaque were 50% diabetic and had an eGFR of 68 ± 19 ml/min/1.73 m^2^ but these features were not different from those associated with fibrous plaque (29%, p = 0.15; and eGFR 76 ± 12 ml/min/1.73 m^2^, p = 0.69).

**Table 2:** Features associated with different plaque type.

Clinical characteristic	Predominant plaque type						
	None (n = 10)	Fibrous (n = 7)	Mean difference (95% CI) p value (fibrous: none)	Lipid (n = 7)	Mean difference (95% CI), p value (lipid: none)	Calcific (n = 10)	Mean difference (95% CI), p value (calcific: none)
Age (years)	65.8 ± 9.5	56.3 ± 9.6	**−9.50 (−19.52 to 0.52) p = 0.06**	63.1 ± 7.4	−2.70 (−11.86 to 6.46) p = 0.54	68.4 ± 9.4	2.6 (−6.28 to 11.48) p = 0.55
Male	10 (100%)	5 (71%)	p = 0.15	7 (100%)	p = 1.00	7 (70%)	p = 0.21
Hypertension	5 (50%)	6 (86%)	p = 0.30	6 (86%)	p = 0.30	9 (90%)	p = 0.14
Systolic BP (mm Hg)	124.0 ± 16.9	119.7 ± 11.3	−4.30 (−19.97 to 11.37), p = 0.57	124.1 ± 9.5	0.10 (−15.0 to 15.3) p = 0.99	120.6 ± 10.0	−3.40 (−16.45 to 9.64, p = 0.59
Diastolic BP (mm Hg)	67.0 ± 13.4	76.6 ± 7.8	9.60 (−2.47 to 21.67) p = 0.11	73.9 ± 7.0	6.90 (−4.95 to 18.75) p = 0.22	70.8 ± 5.8	3.8 (CI −5.90 to 13.50), p = 0.42
Mean arterial BP (mm Hg)	86.1 ± 13.9	91.8 ± 8.0	5.70 (−6.80 to 18.20) p = 0.35	91.6 ± 4.2	5.50 (−6.15 to 17.15) p = 0.33	87.4 ± 3.4	1.30 (−8.21 to 10.81) p = 0.78
Diabetes	0	2 (29%)	p = 0.15	2 (29%)	p = 0.15	5 (50%)	p = 0.62
Smoking history	8 (80%)	5 (71%)	p = 1.00	6 (86%)	p = 1.00	8 (80%)	p = 1.00
eGFR (ml/min/1.73 m^2^)	73 ± 17	76 ± 12	3 (−13.0 to 19) p = 0.69	76 ± 14	3 (−14 to 20) p = 0.71	68 ± 19.1	−5 (−22 to 12) p = 0.54
**Angiography findings**							
LAD stenosis	0	5 (71%)	**p = 0.003**	4 (57%)	**p = 0.02**	9 (90%)	p = 0.54
Diseased vessel score (4 vessels studied)	0.1 ± 0	1.0 ± 1.0	**p = 0.01**	1.7 ± 1.4	**p = 0.002**	2.2 ± 1.3	p = 0.0001
Number of stents	0 ± 0	1.0 ± 1.0	**p = 0.006**	1.0 ± 0.4	**p = 0.0001**	1.9 ± 1.2	p = 0.0001
Plaque thickness	N/A	0.15 ± 0.1		0.16 ± 0.1	0.01 (−0.11 to 0.13) p = 0.85	0.6 ± 3.2	0.45 (−2.15 to 3.05) p = 0.72
Plaque cap thickness	N/A	0.67 ± 3.4		0.35 ± 3.9	−0.32 (−4.58 to 3.94) p = 0.87	0.17 ± 0.13	−0.50 (−2.76 to 1.76) p = 0.64
Plaque arc	N/A	109 ± 49		116 ± 74	7 (−66.1 to 80.1) p = 0.84	71 ± 46	−38 (−87.6 to 11.6) p = 0.12
**Retinal abnormalities**							
Microvascular retinopathy Mild	9 (90%)	6 (86%)	p = 0.44	4 (57%)	p = 0.06	2 (20%)	**p = 0.001**
Moderate	0	1 (14%)		3 (43%)		8 (80%)	
Haemorrhage	0	1 (14%)	p = 0.41	3 (43%)	p = 0.05	7 (70%)	**p = 0.003**
Exudates	0	0	p = 1.00	0	p = 1.00	2 (20%)	**p = 0.47**
CRAE (um)	143.8 ± 10.6	145.4 ± 13.4	1.6 (−10.80 to 14.00) p = 0.79	134.1 ± 17.1	−9.70 (−23.96 to 4.56) p = 0.17	158.4 ± 15.2	**14.60 (2.90 to 26.91) p = 0.02**
CRVE (um)	211.4 ± 10.1	212.5 ± 15.1	1.10 (−11.87 to 14.07) p = 0.86	196.7 ± 31.5	−14.70 (−37.18 to 7.78) p = 0.18	219.3 ± 16.6	7.90 (−5.01 to 20.81), p = 0.21

Subjects with mainly fibrous, lipid or calcific plaque were more likely to have an LAD stenosis, a higher mean diseased vessel score, and an increased mean number of stents than subjects with normal angiograms (Table 2). Subjects with mainly calcific plaque had an LAD stenosis in 90% cases, a higher mean diseased vessel score (p < 0.001), and a greater number of stents (p < 0.001) than those with mainly fibrous plaque.

Average plaque length, thickness, cap thickness and arc angle were not different in subjects with fibrous, lipid or calcific plaque (Table 2).

### Retinopathy features

All subjects with intracoronary plaque had a hypertensive microvascular retinopathy, with mild features in 14 (50%) and moderate in the others (50%) (Fig. 1d–i). Only two individuals had retinal exudates, and both had calcific plaque.

Their overall mean retinal arteriole calibre was 147.6 ± 17.9 µm and mean venular calibre was 213.0 ± 24.9 µm, but the calibre was not different from normal (p = 0.79 and p = 0.86 respectively) (Table 1).

Overall subjects with lipid or calcific plaque were more likely to have a moderate microvascular retinopathy (p = 0.05, p < 0.001 respectively) and haemorrhage (p = 0.05, p = 003 respectively) than those with normal angiography (Table 2).

### Comparison of retinopathy features in subjects with intracoronary plaque or none

Larger arteriole calibre was not associated with older age, male gender, smoking history, diabetes, hypertension, mean arterial pressure or reduced eGFR (p all NS) (Table 3).

**Table 3:** Features associated with smaller and larger retinal arteriole calibre.

Clinical and plaque characteristics	CRAE ≤ 154 µm	CRAE > 154 µm	p value
Patient characteristics (n = 28)	n = 14	n = 14	
Age (mean ± SD, years)	62.7 ± 8.5	62.4 ± 10.7	0.94
Males	10 (71%)	12 (86%)	0.65
Current and former smokers	10 (71%)	11 (79%)	1.00
Cigarette pack years (mean ± SD)	27.3 ± 14.3	28.3 ± 30.1	0.92
Diabetes	4 (29%)	6 (43%)	0.69
Hypertension diagnosis	14 (100%)	10 (71%)	0.10
Systolic BP (mean ± SD, mmHg)	126.4 ± 12.6	120.2 ± 9.1	0.16
Diastolic BP (mean ± SD, mmHg)	73.4 ± 7.9	74.0 ± 5.4	0.80
Mean arterial BP (mean ± SD, mmHg)	91.0 ± 6.3	89.4 ± 5.1	0.47
eGFR (mean ± SD, mL/min/1.73 m^2^)	72.2 ± 15.6	76.6 ± 15.0	0.46
Plaque characteristics (n = 161)	n = 72	n = 89	
**Plaque type**			
Lipid (n = 54)	36 (50%)	18 (20%)	<0.0001
Calcific (n = 75)	22 (30%)	53 (60%)	<0.001
Fibrous (n = 32)	14 (19%)	18 (20%)	1.00
Plaque length (mm)	3.2 ± 2.7	2.9 ± 2.7	0.35
Plaque thickness (mean ± SD, mm)	0.37 ± 0.17	0.50 ± 0.65	0.33
Plaque cap thickness (mm, lipid plaques only) (n = 54)	0.20 ± 0.09 (n = 36)	0.18 ± 0.08 (n = 18)	0.39
Arc (°)	109 ± 74	103 ± 68	0.54

However larger arteriole calibre was associated with calcific plaque (p < 0.001) and smaller calibre with lipid plaque (p < 0.0001). Arteriolar calibre was also not associated with plaque length, thickness, cap thickness or maximum arc. Venular calibre did not differ with plaque type.

## Discussion

Many studies have demonstrated that retinal microvascular abnormalities predict cardiac ischemic events. Here we examined a cohort of individuals undergoing intracoronary OCT for any association between retinal vascular abnormalities and plaque features. All subjects with coronary artery plaque had a mild or moderate microvascular retinopathy, and were more likely to have moderate microvascular disease than those with no plaque. Moderate retinopathy was also more common with lipid or calcific plaque. Arteriole calibre was larger in individuals with calcific plaque but was not associated with diabetes or a smoking history.

In this cohort, intracoronary plaque was more common with hypertension, diabetes and a higher mean diastolic BP. Not unexpectedly, plaque was also more common where there was LAD stenosis >70%, an increased mean diseased vessel score and increased mean stent number.

Subjects with intracoronary plaque were more likely to have a microvascular retinopathy, and in particular, a moderate-grade retinopathy, compared with those with no plaque. Subjects with intracoronary plaque had more hypertension, diabetes, and a higher mean diastolic BP than those without. Diastolic BP is especially important as a risk factor for cardiac disease because it represents the BP for two-thirds of the cardiac cycle.

Subjects with fibrous plaque had only a mild microvascular retinopathy, and their arteriole and venular calibre were normal. This was consistent with fibrous plaque being the earliest phase of plaque development and associated with the lowest diseased vessel score of all the subjects with plaque.

Subjects with lipid plaque had more LAD stenosis, a higher mean diseased vessel score and more stents. They also were more likely to have a moderate retinopathy but arteriole and venular calibre were normal. When all lipid plaques were taken into account though, vessel calibre was still smaller.

Subjects with calcific plaque were even more likely to have LAD stenosis, and a higher diseased vessel score and more stents. They were also more likely to have a moderate retinopathy. The only two subjects with retinal hard exudates both had calcific plaque on OCT. Subjects with calcific plaque also had a larger arteriole calibre than normal. The increased calibre was confirmed when all plaques were taken into account.

These results suggest increasing severity of microvascular retinopathy from fibrous to lipid to calcific plaque. However arteriole calibre did not increase progressively. Arteriole calibre was normal for fibrous and lipid plaque, and increased for calcific plaque. Increase in arteriole calibre occurs with diabetes and smoking, but the increased calibre is unexplained here because too few subjects were studied. We have shown previously that the vasoactive agents (radio-contrast, anginine, verapamil) used during the angiography do not affect retinal vessel calibre. Interestingly previous reports have described a correlation of cardiac events with both smaller arterioles and larger venules.

Microvascular calibre is complex with the same vascular risk factors as coronary artery disease, namely male gender, age, hypertension, renal failure, smoking, diabetes, obesity and inflammation. Typically arteriole and venular calibre are interdependent. The difference in calibre for lipid and calcific plaque may occur because of the various risk factors in different populations, such as smaller arteriole calibre in older men, with hypertension, or renal impairment, and larger venules in those with obesity, diabetes or a smoking history.

In summary, this study found that individuals with intracoronary plaque were more likely to have hypertension and to have a moderate microvascular retinopathy than those with no plaque. Half of the individuals with calcific plaque had diabetes and larger retinal arterioles compared with those with no plaque where none had diabetes. The small cohort size meant that it was not possible to exclude the diagnoses of hypertension and diabetes as the causes of these respective features.

This study’s major strengths were its novelty and the robust measurements used to assess retinopathy and microvascular calibre. Its limitations were its cross-sectional nature and the small sample size. Recruitment was limited because coronary angiography can only be undertaken for clinical indications and intracoronary OCT is expensive. While plaque was only evaluated in the culprit vessels, this study excluded mixed types and examined predominant plaque types separately. While the peripheral retinal view was limited it was still sufficient to grade the retinopathy and more than sufficient to measure vessel calibre.

This study has demonstrated for the first time an association between systemic small vessel disease and the presence and type of intracoronary plaque. The demonstration of a moderate microvascular retinopathy reminds us of the risk of poorly-controlled hypertension in cardiac disease and the need for optimizing control. The presence of diabetes in half of those with calcific plaque may reflect diabetes-associated vascular risk. The role of retinal imaging needs to be explored further both in identifying the presence of intra-coronary artery plaque as well as its likely composition.

## Data Availability

All data is deidentified and available from the corresponding author.
